# Low-Dose Vitamin C-Based Electroporation of Solid Tumors: A New Area in Non-Cytotoxic Electrochemotherapy

**DOI:** 10.3390/biomedicines14040936

**Published:** 2026-04-20

**Authors:** Seyed Mojtaba YazdanParast, Navid Manoochehri, Mohammad Abdolahad

**Affiliations:** 1 Nano Electronic Center of Excellence, Nano Bio Electronic Devices Lab, School of Electrical and Computer Engineering, University of Tehran, Tehran P.O. Box 14395/515, Iran; sm.yazdanparast@ut.ac.ir (S.M.Y.); manoochehri.n@ut.ac.ir (N.M.); 2 UT and TUMS Cancer Electronics Research Center, Tehran University of Medical Sciences, Tehran P.O. Box 14395/515, Iran

**Keywords:** electrochemotherapy, reversible electroporation, vitamin C, variable plate electrode, tumor growth inhibition

## Abstract

**Background**: Electrochemotherapy enhances the intracellular delivery of anticancer agents through electroporation but is traditionally limited to cytotoxic drugs associated with significant side effects. Vitamin C (ascorbic acid) exhibits selective anticancer activity when accumulated at high intracellular concentrations; however, its therapeutic application is restricted by poor membrane permeability and rapid systemic clearance. **Methods**: In this study, we investigated whether reversible electroporation, applied using a custom-designed variable plate electrode system designed to deliver a uniform electric field, could potentiate the antitumor efficacy of low-dose vitamin C. Numerical simulations were performed to optimize electrode spacing and stimulation voltage, suggesting homogeneous electric field coverage throughout the tumor volume. The proposed approach was evaluated in vitro using MDA-MB-231 and 4T1 breast cancer cell lines and in vivo in a 4T1 murine breast cancer model. **Results:** Low-dose vitamin C alone produced minimal cytotoxic effects, whereas its combination with electroporation significantly reduced cell viability and increased apoptotic and necrotic cell death in vitro. In vivo, vitamin C–assisted electrochemotherapy resulted in pronounced tumor growth suppression, with tumor volumes reduced to approximately 0.34-fold of baseline by day 15, accompanied by decreased proliferation and marked tissue disruption. **Conclusions:** These findings demonstrate that uniform-field reversible electroporation markedly enhances the intracellular delivery and antitumor activity of low-dose vitamin C, supporting this technology-driven strategy as a promising, low-toxicity alternative to conventional chemotherapeutic agents in electrochemotherapy for solid tumors.

## 1. Introduction

Electrochemotherapy is a technique that administers chemotherapy directly into cancerous cells. This method combines chemotherapy—either injected directly into the tumor or administered through the bloodstream—with an electrical impulse, which enhances the penetration of the chemotherapy into the cancer cells through a process known as electroporation. This treatment is designed specifically for solid tumors using cytotoxic agents that are poorly absorbed, such as bleomycin or cisplatin [1,2]. While these drugs can be effective, they are also associated with various side effects [3]. In cases of frequent disease recurrence, the reuse of these medications may be limited due to the potential risk of developing pulmonary fibrosis after reaching the lifetime recommended dosage [4]. Consequently, pursuing effective drugs with fewer side effects remains a priority of great interest.

Vitamin C, as an antioxidant, is essential in numerous cellular functions. It is vital for various metabolic processes, including maintaining the extracellular matrix and aiding effective communication between the cell’s genome and proteome [5]. A higher metabolic activity in a region corresponds to a higher concentration of vitamin C in that region [6].

Vitamin C can penetrate cells only gradually, mainly through two mechanisms: passive diffusion and active transport mediated by specialized sodium-dependent vitamin C transporters, SVCT1 and SVCT2 [6]. Analyses indicate that achieving a steady intracellular level of ascorbate depends only weakly on its plasma concentration and typically requires several days of exposure to attain the levels associated with therapeutic or anticancer activity [7]. In extracellular compartments, ascorbate concentrations are tightly maintained, and any surplus is rapidly eliminated from the body [8]. The spatial distribution of intracellular ascorbate is maintained by dynamic fluxes that must be continuously adjusted to meet changing metabolic demands [9]. Under normal physiological conditions, the intracellular concentration of vitamin C is primarily regulated by sodium-dependent vitamin C transporters (SVCTs), whose active uptake is counterbalanced by passive transport across the plasma membrane. Passive diffusion follows Fick’s law and is proportional to the transmembrane concentration gradient and the membrane potential.

At steady state, the net transmembrane flux of vitamin C is zero, such that active uptake, passive efflux, and intracellular metabolism are in balance:

T_active_ − T_passive_ − T_metabolism_ = 0

which can be rearranged as:

n_svct1_ · a_svct1_ · C_out_ + n_svct2_ · a_svct2_ · C_out_ − T_passive_ = T_metabolism_

In this Equation, the first two terms describe the contributions of SVCT1 and SVCT2 to active vitamin C uptake, where n_svct1_ and n_svct2_ denote the number or surface density of each transporter and a_svct1_ and a_svct2_ represent their respective transport activities. *C*_out_ is the extracellular vitamin C concentration. The term T_passive_ represents passive efflux opposing active uptake, while T_metabolism_ accounts for intracellular metabolic consumption of vitamin C [10]. Because the membrane permeability of vitamin C is extremely low (≈10^−8^ cm/s), passive diffusion across the lipid bilayer is effectively negligible. Consequently, when intracellular ascorbate concentrations rise, they cannot decrease rapidly, as diffusion is the only known pathway for its efflux, and this process is too slow to significantly reduce intracellular levels [11].

**Figure 1:**
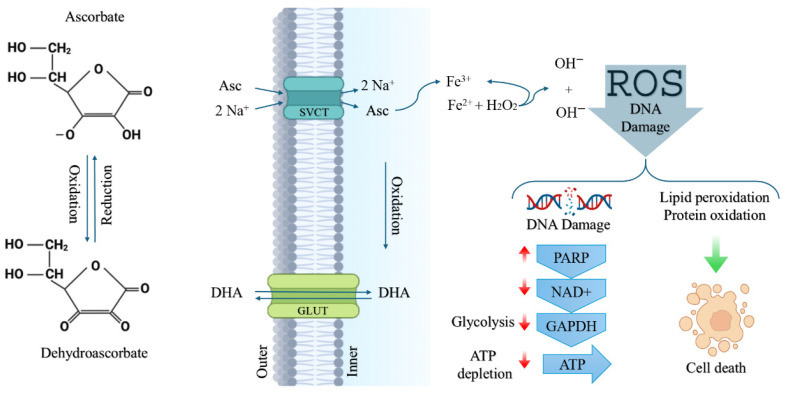
Overview of the cellular actions and anticancer mechanisms of vitamin C. In physiological environments, vitamin C is present predominantly in two interchangeable forms—the reduced vitamin C (Asc) and the oxidized dehydrovitamin C (DHA)—which differ by the transfer of two electrons and two protons. Cellular uptake of ascorbate occurs primarily through SVCTs, resulting in steep intracellular concentration gradients, while passive diffusion remains minimal due to its limited membrane permeability. When intracellular ascorbate becomes sufficiently elevated to reduce Fe^3+^ to Fe^2+^, the labile iron pool expands, enhancing ROS generation and promoting ferroptotic cell death. Pharmacologic vitamin C exerts selective cytotoxicity in cancer cells through ROS-mediated mechanisms. Extracellular Asc undergoes oxidation via Fenton chemistry, producing hydrogen peroxide (H_2_O_2_), which enters cancer cells through aquaporins or passive diffusion. Asc is taken up by SVCT2, while DHA is transported through GLUT1 and subsequently reduced back to Asc at the expense of intracellular glutathione (GSH). GSH depletion, combined with increased intracellular H_2_O_2_, results in DNA damage, lipid and protein oxidation, PARP activation, NAD^+^ depletion, inhibition of GAPDH, glycolytic collapse, decreased ATP production and eventual cell death. This figure was created using BioRender.com, with modifications inspired by [25,26,27,28].

Ascorbate is widely acknowledged as nontoxic; however, when utilized in tissue cultures at concentrations of just a few mM, it has proven to kill cancer cells. This undeniable evidence underscores the vulnerability of cancerous cells to elevated ascorbate levels, indicating its potential as a powerful tool in cancer treatment. Ascorbate’s therapeutic activity does not necessarily require sustained elevation of its concentration in the bloodstream, particularly when increased plasma levels persist for less than one hour [12]. Membrane transport systems are primarily designed to accumulate ascorbate within the cytoplasm, resulting in limited cellular capacity to counteract excessive intracellular levels. Reduction in intracellular ascorbate can occur mainly through passive diffusion across the plasma membrane or through its oxidation to dehydroascorbate (DHA). Once generated, DHA may exit the cell via glucose transporters (GLUTs) or be rapidly reduced back to ascorbate through intracellular metabolic reactions. As a result, oxidation alone does not substantially diminish cytoplasmic ascorbate levels [13]. Research suggests that elevated levels of ascorbate can significantly increase the amount of labile iron in the cytoplasm. This surge in iron triggers a dramatic rise in reactive oxygen species, ultimately resulting in cell death through a process known as ferroptosis [14,15]. The cellular and anti-cancer mechanisms of vitamin C are illustrated in Figure 1.

High-dose ascorbate administration is increasingly recognized for its therapeutic potential in oncology, largely because reactive oxygen species (ROS) are fundamental drivers of both tumor progression and its response to treatment. The safety profile and potential toxicity of vitamin C regimens have been extensively documented across various studies [16,17,18,19,20,21,22,23,24]. High doses of vitamin C might cause diarrhea, nausea, vomiting, heartburn, stomach (abdominal) cramps, and headache. But these possible side effects are much lower than using a chemotherapy drug. In contrast to bleomycin, vitamin C does not produce a comparable level of cytotoxic effect when used under similar conditions.

Due to the slow entry of vitamin C into cells, it is essential to maintain a high concentration for an extended period to allow for the accumulation of vitamin C. This article presents a new model for introducing ascorbate into cells using the electroporation method.

## 2. Materials and Methods

### 2.1. Electroporation Parameters and Pulse Delivery

Electroporation was performed using a custom pulse generator delivering square-wave electric pulses. For each treatment, eight pulses were applied with a pulse width of 100 µs at a frequency of 5 kHz. In the in vivo experiments, electroporation was delivered approximately 1 min after intratumoral injection of the vitamin C solution to allow initial distribution of the agent within the tumor tissue prior to membrane permeabilization. For in vitro electroporation experiments, cells were suspended in phosphate-buffered saline (PBS) to provide a low-conductivity medium and minimize electrical arcing during pulse delivery. Electroporation pulses were applied at 400 V using a 4 mm gap cuvette, corresponding to an electric field strength of 1000 V/cm.

### 2.2. Electrode Design, Distance Sensing, and Voltage Synchronization

A non-invasive electrode was designed for electrical stimulation of treatment regions and modeled using SolidWorks 2024 (Dassault Systèmes, Vélizy-Villacoublay, France). The conductive elements consisted of medical-grade 316 stainless steel blades (0.5 mm thickness), selected for their biocompatibility and suggested capacity to deliver a uniform electric field across the treatment area without requiring frequent repositioning.

The electrode body comprised two polylactic acid (PLA) components fabricated by 3D printing (UltiMaker, Utrecht, The Netherlands). The completed electrodes were sterilized using hydrogen peroxide plasma prior to use.

To enable tumor-size-adaptive electroporation, the electrode system was equipped with an integrated mechanical–electrical distance-sensing mechanism. The inter-electrode separation was continuously measured using a miniaturized linear potentiometer embedded within the probe structure. Mechanical adjustment of the electrode spacing resulted in a proportional change in potentiometer resistance, which was used to calculate the real-time distance between the two conductive plates.

As the operator adjusted the electrode spacing to match the tumor thickness, the linear potentiometer functioned as a variable resistor, generating a voltage signal proportional to electrode displacement. This signal was processed by the control unit to compute the inter-electrode distance with sub-millimeter resolution. The measured distance was then used to dynamically determine the minimum required applied voltage necessary to maintain a constant therapeutic electric field strength (V/cm) within the tumor tissue.

By directly coupling electrode spacing to voltage selection, the system suggested the achievement of the electroporative threshold without the application of excessive absolute voltage. This closed-loop distance–voltage synchronization eliminates the need for empirical voltage escalation commonly employed in fixed-geometry electroporation systems and reduces the risk of unintended electric field exposure in surrounding healthy tissues. The integration of adjustable electrode geometry with real-time distance sensing significantly improves treatment safety, reproducibility, and precision.

### 2.3. Simulation of the Electric Field Distribution

The spatial profiles of electric potential and field strength were modeled using the AC/DC physics interface in COMSOL Multiphysics version 6.4 (COMSOL Inc., Burlington, MA, USA). The computational model included the electrode geometry, the tumor tissue domain, the surrounding normal tissue, and an outer ambient air domain to approximate the experimental configuration. Electrical conductivity and relative permittivity values were assigned to each domain according to literature data. The Electric Currents interface was used to calculate the electric potential and electric field distributions throughout the model. Current conservation was applied in all domains. Electrical insulation boundary conditions were imposed on all exterior boundaries of the model, corresponding to zero normal current flow across the outer surfaces. Fixed electric potentials were assigned to the electrode surfaces to reproduce the applied stimulation conditions, and the initial electric potential was set to zero in all domains. The computational domain was discretized using tetrahedral finite elements in the volumetric domains and triangular elements on the boundary surfaces. Manual mesh refinement was applied near the electrodes and within the tissue domains to improve numerical accuracy in regions with steep electric field gradients, while a coarser mesh was used in the outer air domain to reduce computational cost. The final mesh consisted of 3,806,006 tetrahedral elements and 249,229 triangular elements, with a minimum mesh size of 0.02 mm in the vicinity of the electrodes. The simulation model was developed to assess treatment efficiency and to determine suitable voltage parameters and electrode spacing. Therefore, the application of the electrode for typical tumor size of a 1 cm was modeled and simulated. As expected, a uniform field of 0.8 to 1 kV/cm was applied to the treated area by placing the tumor between two electrodes. The applied voltage and resulting electric field distribution between the electrodes were calculated using the relations E = −∇V and J = σE, where E is the electric field intensity, V is the electric potential, J is the current density, and σ is the electrical conductivity of the medium.

Material properties were assigned to all computational domains to accurately represent their electrical behavior during simulation. The electrical conductivity and relative permittivity of the biological tissues, including the 4T1 tumor and surrounding healthy tissue, were defined based on values reported in the literature [29,30]. As summarized in Table 1, the tumor tissue was modeled with a higher electrical conductivity than healthy tissue, reflecting its increased ionic content, while both tissues were assigned high relative permittivity values consistent with their water-rich composition. Non-biological materials were also included in the model: air and polylactic acid (PLA) were treated as electrical insulators with very low conductivities and low relative permittivities, whereas conductive hydrogels were assigned high conductivity and permittivity to represent their role in facilitating current distribution. Stainless steel (316) was modeled as a highly conductive material with the unit of relative permittivity, corresponding to its metallic nature. These material assignments enabled a realistic simulation of electric field distribution across heterogeneous biological and structural domains.

### 2.4. Cell Culture

The 4T1 and MDA-MB-231 breast cancer cell lines were obtained from the National Cell Bank of the Pasteur Institute of Iran. After thawing, cells were transferred to culture flasks (SPL Life Sciences, Gyeonggi-do, Republic of Korea, Cat. No. 70025) and grown in a medium consisting of 89% RPMI-1640 (Gibco, Thermo Fisher Scientific, Grand Island, NY, USA, Cat. No. 11875093), supplemented with 10% fetal bovine serum (FBS, Gibco, Thermo Fisher Scientific, Grand Island, NY, USA, Cat. No. A5670701) and 1% penicillin–streptomycin (100×, Gibco, Thermo Fisher Scientific, Grand Island, NY, USA, Cat. No. 15140122). Cultures were maintained at 37 °C in a humidified incubator with 5% CO_2_. The cell culture medium was refreshed daily, and prior to conducting any experiments, the cells were thoroughly assessed to ensure they were free of biological contamination.

### 2.5. Measurement of Intracellular Reactive Oxygen Species (ROS)

Intracellular reactive oxygen species (ROS) generation was evaluated using a ROS detection kit (Beyotime Biotechnology, Shanghai, China) according to the manufacturer’s instructions. Briefly, MDA-MB-231 breast cancer cells were seeded in six-well plates at a density of 7 × 10^5^ cells per well and allowed to adhere overnight. The cells were then subjected to the designated treatments. To evaluate the potential contribution of extracellular hydrogen peroxide, two additional experimental groups were included in which catalase (from bovine liver; Sigma-Aldrich, Merck, St. Louis, MO, USA, Cat. No. C9322) was added to the PBS treatment solution at a final concentration of 20 µg/L to enzymatically decompose extracellular H_2_O_2_. During treatment, cells were maintained in the PBS-based solution containing the respective reagents for 15 min. After completion of the treatment, the PBS solution was removed and replaced with fresh culture medium. ROS production was then assessed using 2′,7′-dichlorodihydrofluorescein diacetate (DCFH-DA). Cells were incubated with 5 µM DCFH-DA in 1 mL medium at 37 °C for 20 min in the dark. Following incubation, the cells were washed with phosphate-buffered saline (PBS) to remove excess dye. The fluorescence signal, indicating intracellular ROS generation, was observed and recorded using a fluorescence microscope (OPTIKA Srl, Ponteranica, Italy).

### 2.6. Tumor Induction and Experiments in Mouse Model

Female BALB/c mice (4–6 weeks old, weighing approximately 18–22 g) were obtained from the Pasteur Institute of Iran. The animals were maintained in a pathogen-free animal facility under controlled environmental conditions (22–25 °C, 50–60% relative humidity) with a 12 h light/12 h dark cycle and unrestricted access to food and water. Throughout the study, the mice were observed daily to monitor overall health status and tumor development. For tumor xenograft experiments, mice were inoculated with tumor cells and, after tumor establishment, were randomly assigned to the experimental groups (*n* = 5 per group).

To minimize measurement bias, tumor measurements and data collection were performed by an investigator blinded to treatment allocation. Humane endpoints were predefined in accordance with institutional ethical guidelines and included tumor diameter exceeding 15–20 mm, tumor ulceration, or signs of severe distress or weight loss greater than 20%. Animals reaching these criteria were humanely euthanized. To control for potential mechanical effects, a vitamin C–only group was included in which tumors received the same intratumoral injection without electroporation pulses. Electroporation was performed using non-invasive plate electrodes placed externally on the skin surface, avoiding additional mechanical injury to the tumor.

Survival outcomes were analyzed using Kaplan–Meier survival curves (*n* = 10 group size).

In Tumor induction in the animal model was performed using the 4T1 breast cancer cell line. Cells were first harvested from culture flasks by removing the growth medium and gently washing the flask surfaces with 1× phosphate-buffered saline (PBS; Gibco, 10010023). A 0.25% trypsin–EDTA solution was then added to detach the adherent cells. After approximately 3 min of incubation, the enzymatic reaction was neutralized by adding culture medium containing fetal bovine serum, and the resulting cell suspension was collected in conical tubes (SPL, 50015). The samples were centrifuged at 1500 rpm for 5 min, after which the supernatant containing the trypsin solution was discarded.

To eliminate any remaining trypsin–EDTA and serum components before injection, the cell pellet was resuspended in PBS and washed twice through additional centrifugation steps. For tumor establishment, 1 × 10^6^ 4T1 cells were prepared in 0.1 mL PBS and administered subcutaneously into the flank of each mouse. Tumor development was monitored using ultrasound imaging, and tumor size was estimated using an equation approximating the volume of an ellipsoid before surgical intervention.

$$Tumor\;Volume=\frac{4}{3}\times \pi \times (\frac{Depth}{2})\times (\frac{Width}{2})\times (\frac{Length}{2})$$

Based on previous in vivo studies, vitamin C was administered by intratumoral injection using a strictly volume-limited approach (40–50 µL per tumor). Tumor size was first estimated from ultrasound-derived tumor volume measurements. To maintain consistent local exposure while avoiding excessive intratumoral pressure, a fixed injection volume was used. Vitamin C was prepared at a concentration of 100 mg/mL, resulting in an intratumoral delivery of approximately 4–5 mg vitamin C per tumor, depending on tumor volume. No macroscopic tumor rupture or leakage was observed following injection. Specifically, one study in oral squamous carcinoma demonstrated enhanced therapeutic effects of cisplatin when combined with high-dose vitamin C (4 g/kg IP, twice daily) [31]. Similarly, another study in pancreatic cancer reported significant tumor growth inhibition when gemcitabine was administered alongside high-dose vitamin C at the same dosage [32]. Pharmaceutical-grade vitamin C was obtained from Darou Pakhsh Pharmaceutical Mfg. Co., Tehran, Iran (500 mg/5 mL ampoules; intended for I.M. or I.V. injection). The solution was stored at 2–8 °C, protected from light, and freshly used for intratumoral injection and in vitro experiments to minimize oxidation.

Animals were anesthetized via intraperitoneal injection of ketamine (Bremer Pharma GmbH, Warburg, Germany; 100 mg/kg) and xylazine (Sedaxyl^®^, Kela Veterinaria, Hoogstraten, Belgium; 10 mg/kg). Anesthesia was maintained during the procedure by administering supplemental doses corresponding to approximately 10–25% of the initial ketamine/xylazine dose as required.

Following electrochemotherapy (ECT), mice received standard post-procedural care to minimize pain and distress. Analgesia was provided using buprenorphine (0.05–0.1 mg/kg, subcutaneously) for up to 72 h, with meloxicam (1–5 mg/kg, subcutaneously) administered once daily as needed. Antibiotics were not routinely used and were administered only in cases of suspected infection or severe skin damage. Animals were closely monitored daily for general health, body weight, behavioral changes, and tumor site condition in accordance with approved ethical guidelines.

### 2.7. Cell Culture Treatment with Vitamin C

Pharmacokinetic studies show that taking 1.25 g of vitamin C daily results in average peak plasma vitamin C levels of 135 micromol/L, approximately twice the levels achieved by consuming 200–300 mg per day from foods high in vitamin C [8]. Pharmacokinetic modeling suggests that taking doses of vitamin C up to 3 g every 4 h would result in peak plasma levels of merely 220 micromol/L [8]. A significant distinction exists between orally administered vitamin C (OC) and high-dose intravenous vitamin C (IVC). Orally administered vitamin C achieves peak plasma concentrations that do not exceed 220 μmol/L, while high-dose IVC can raise plasma concentrations to the millimolar level (≥15 mmol/L). This elevated concentration is crucial for eliciting cytotoxic effects in cancer cells, as evidenced by preclinical research. The researchers showed that ascorbate concentrations of 500 µM and above induced cytotoxicity in MDA-MB-231 cells, while 1 mM produced clear cytotoxic effects in MCF7 and EO771 cells, evidenced by morphological changes and loss of adherence. This response aligns with the well-established generation of H_2_O_2_ at high ascorbate levels, with significant toxicity typically occurring around 1 mM [33]. Based on these findings, a concentration of 1 mmol/L vitamin C was identified as a suitable therapeutic level for effective treatment of the cell line. Additionally, it is important to note that vitamin C has a relatively brief half-life of less than 2 h in the bloodstream, which is primarily due to its rapid clearance by the kidneys [34].

Cells were harvested from culture flasks by first removing the growth medium and gently rinsing the flask surface with 1× phosphate-buffered saline (PBS; Gibco, 10010023). A 0.25% trypsin–EDTA solution was then added to detach the adherent cells. After approximately 3 min of incubation, the enzymatic reaction was neutralized by adding culture medium supplemented with fetal bovine serum. The resulting cell suspension was collected in conical tubes (SPL, 50015) and centrifuged at 1500 rpm for 5 min. The supernatant containing the trypsin solution was discarded. Cell pellets were resuspended in phosphate-buffered saline (PBS; Gibco, Thermo Fisher Scientific, Grand Island, NY, USA; Cat. No. 10010023). For the vitamin C treatment groups, vitamin C was dissolved in PBS to reach the desired concentration. The control group was resuspended in PBS without vitamin C. Cell suspensions were transferred into 4 mm electroporation cuvettes. Electroporation pulses were applied to the designated electroporation groups. The vitamin C–only group was placed in the cuvettes but did not receive electrical stimulation. Following electroporation, the cells remained in the PBS solution for 15 min to allow membrane resealing and interaction with the treatment conditions. After incubation, the samples were centrifuged at 1500 rpm, the PBS solution was removed, and the cells were resuspended in RPMI culture medium for subsequent incubation and analysis.

The effects of electroporation alone can be clearly evaluated using this methodology in comparison to the effects of vitamin C alone. Furthermore, it is crucial to emphasize that the pores formed during the electroporation procedure usually seal in around fifteen minutes [35].

Each treatment group consisted of five wells for every experiment. Each experiment was repeated at least five times. The viability of the cells was carefully evaluated using live/dead (AO/PI) staining in conjunction with a flow cytometric assay to assess apoptosis and necrosis.

### 2.8. Cytopathology Assay

The process of tissue handling for cytopathology analysis begins with dehydrating the samples. Tissue dehydration was carried out by sequential exposure to graded ethanol solutions (Merck, Sigma-Aldrich, St. Louis, MO, USA; Cat. No. 100983). The samples were transferred through ethanol concentrations of 70%, 85%, 96%, and finally 100%, remaining in each solution for 15 min. After dehydration, the tissues were cleared in xylene (Merck, 108297), which is compatible with both ethanol and paraffin. Subsequently, the processed tissues were embedded in paraffin blocks. Thin sections with a thickness of approximately 5 µm were then prepared from the paraffin blocks using a microtome.

After sectioning, the paraffin slices were floated on a distilled water bath maintained at 40 °C and then carefully mounted onto glass slides. The slides were subsequently placed in an oven at 60 °C to improve tissue adhesion to the slide surface and to assist in removing residual paraffin.

For hematoxylin and eosin (H&E) staining, the mounted sections were first deparaffinized by immersion in xylene for 2 min. Rehydration was then carried out by passing the slides through decreasing concentrations of ethanol (100% followed by 96%), with each step lasting 2 min. The sections were rinsed in water for 2 min and stained with hematoxylin for 5 min, followed by a 5 min wash in running water. Differentiation was performed using acid-alcohol prepared from 1% HCl (Merck, 100317) in 70% ethanol, after which the slides were rinsed again with water. Bluing of the nuclei was achieved using saturated lithium carbonate solution (Merck, 105680), followed by another water wash.

Before counterstaining, the sections were briefly immersed in 96% ethanol for 1 min. Eosin staining was then carried out using 1% Eosin Y for 10 min. After staining, dehydration was completed by sequential immersion in 95% and 100% ethanol for 1 min each. The slides were finally cleared in xylene for 2 min and coverslips were mounted using Entellan mounting medium (Merck, 107960).

Microscopic examination of the stained sections was performed using a light microscope (OPTIKA Srl, Ponteranica, Italy), and images were recorded with a digital camera (Canon EOS 2000D, Canon Inc., Tokyo, Japan) Histopathological evaluation was conducted by a qualified pathologist.

### 2.9. Immunohistochemistry Assay

Formalin-fixed, paraffin-embedded tissue sections were used for immunohistochemical analysis. The staining procedures for E-cadherin (Dako/Agilent Technologies, Glostrup, Denmark; dilution 1:100), Ki-67 (Abcam, Cambridge, UK; dilution 1:200), p53 (Dako/Agilent Technologies, Denmark; dilution 1:100) and β-catenin (Abcam, Cambridge, UK; dilution 1:200) have been previously described in the literature. Briefly, antigen retrieval was carried out by autoclaving the sections at 121 °C for 15 min in 0.01 mol/L citrate buffer (pH 6.0). To block endogenous peroxidase activity, the slides were incubated with 3% hydrogen peroxide for 30 min at room temperature.

Non-specific binding sites were then blocked by incubating the sections with 10% normal goat serum for 1 h. The samples were subsequently incubated overnight at 4 °C with mouse monoclonal primary antibodies directed against the respective target proteins. After primary antibody incubation, the streptavidin–biotin detection system with horseradish peroxidase was applied using the Histofine SAB-PO (M) immunohistochemical staining kit (Nichirei, Tokyo, Japan) according to the manufacturer’s protocol to visualize antigen–antibody complexes. Nuclear counterstaining was finally performed using Mayer’s hematoxylin.

### 2.10. Statistical Methods and Data Analysis

At least three independent experiments were performed for each assay. Data were analyzed using GraphPad Prism 8 (GraphPad Software, Boston, MA, USA) and are presented as mean ± standard deviation (SD). For comparisons involving more than two experimental groups, statistical significance was determined using one-way analysis of variance (ANOVA) followed by Tukey’s multiple comparisons post hoc test. Longitudinal tumor growth data were analyzed using one-way repeated-measures ANOVA with treatment group as the between-subjects factor and time as the within-subjects factor. Survival analysis was performed using Kaplan–Meier survival curves. A *p*-value < 0.05 was considered statistically significant.

### 2.11. Ethics Statement

All animal procedures were conducted in accordance with the guidelines of the Ministry of Health and Medical Education for the care and use of laboratory animals (IR.ACECR.REC). Ethical approval for this study was obtained from the Research Ethics Committee of the Motamed Cancer Institute, Academic Center for Education, Culture and Research (IBCRC.REC.1400.022).

## 3. Results and Discussion

### 3.1. Pulse Generator Design Considerations and Fabrication

**Figure 2:**
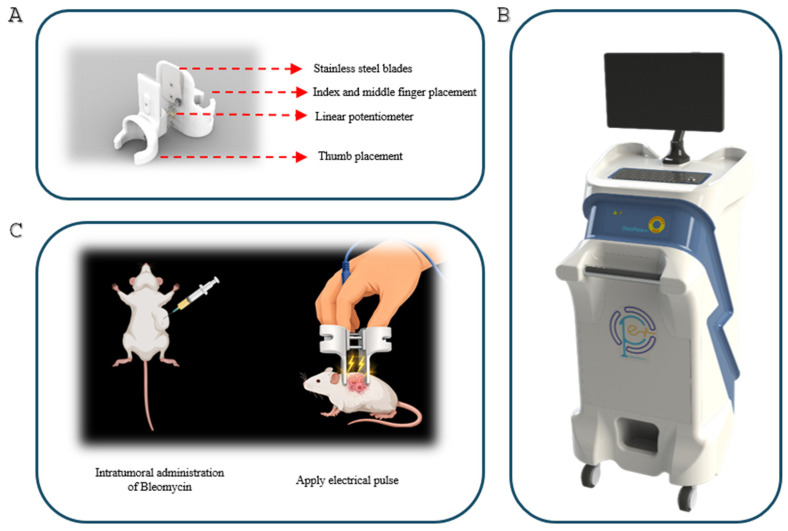
Schematic overview of the experimental setup. (**A**) Electroporation probe. (**B**) Pulse generator (OncoPore). (**C**) Intratumoral administration of vitamin C followed by electroporation. Electrical stimulation consisted of 8 monopolar pulses with an amplitude of 1000 V/cm.

The Oncopore electroporation system (Oncopore, Tehran, Iran) is specifically engineered to electroporate tumors (Figure 2B) efficiently. The device is capable of delivering electrical pulses with adjustable amplitudes ranging from 100 to 1000 volts in either monopolar or bipolar configurations. The duration of each pulse can be set anywhere between 2 µs and 50 ms. This system includes a voltage multiplier and a full-bridge circuit that can deliver the necessary electrical stimulations with feedback circuits for current and voltage. Electric pulses were delivered at a field intensity of 1000 V/cm, with an amplitude exceeding 1000 V and a pulse length of 100 µs. The treatment regimen included eight electric pulses per stimulation, generated at a frequency of 5000 Hz. This system is designed to work with various electrodes and probes; however, this article discusses only plate electrodes spaced 10 mm apart (Figure 2A).

### 3.2. Design and Manufacturing of Electrodes

The variable plate electrode was designed and fabricated for easy electroporation of superficial tumors. This probe does not provide a suitable surface for tissue-electrode contact; hence, conductive gel plays a vital role in ensuring a seamless connection between the blade and the tissue, significantly enhancing the electrode’s overall performance and effectiveness.

The design of the adjustable-plate electrode is shown in Figure 2A. The electrode housing was produced using fused deposition modeling (FDM)–based 3D printing. Polylactic acid (PLA) was selected as the primary material for the main structure due to its biocompatibility and adequate mechanical stability.

The probe consists of two rigid body segments mechanically coupled by two parallel cylindrical elements: a variable linear potentiometer and a conductive cylindrical shaft. The linear potentiometer provides continuous, real-time measurement of the inter-electrode displacement, while the conductive shaft serves as both a structural guide and an electrical pathway for delivery of the electroporation pulses. Together, these cylindrical components constrain relative motion to a single translational axis, preserve parallel alignment of the electrodes, and enhance overall mechanical stability of the probe during operation.

Two blades made of medical-grade 316 stainless steel served as the conductive components of the electrode. This material was selected not only for its excellent biocompatibility but also for its high electrical conductivity, which reduces voltage loss across the electrode blades during pulse delivery. The blade edges were polished to allow smooth movement of the electrode across the tumor surface while minimizing mechanical irritation or damage to the underlying tissue during stimulation.

The lower section of the electrode assembly secures the blades to the mounting structure and maintains a fixed distance between them. The upper portion of the mount was designed with a dual function: it provides a convenient grip for handling the compact electrode and encloses the region where the blade extensions are connected to copper wires that link the electrode to the electroporation device.

In the treatment procedure, chemotherapeutic agents were first administered directly into the tumor (Figure 2C). Electroporation pulses were then applied immediately following the intratumoral injection. Tumor electroporation was performed using the Oncopore electroporation system (Oncopore, Iran) (Figure 2B).

### 3.3. Simulation of Electric Field Distribution Using a Variable-Distance Electroporation Probe

A variable-distance plate electrode was designed to enable precise control of the electric field during electroporation treatment. The probe consists of two opposing flat plate electrodes whose separation can be dynamically adjusted, allowing the electric field intensity to be maintained at a constant and therapeutically effective level despite variations in tissue geometry or target depth. This adjustable configuration is particularly advantageous for electroporation-based therapies, where uniform electric field distribution is important and may help promote reversible membrane permeabilization while minimizing tissue damage. Electric potential and electric field distributions were analyzed using numerical simulations performed with the AC/DC module of COMSOL Multiphysics 6.4. A computational framework was developed to assess the effectiveness of the plate electrode for tumor treatment, in which the tumor was located between the electrodes and subjected to applied electrical stimulation. The numerical model was constructed to represent a simplified but physiologically relevant electroporation scenario. The geometry consisted of a spherical tumor region with a diameter of 1 cm located on healthy tissue domain, which served as the electrical background medium. This configuration was selected to mimic subcutaneous solid tumors commonly used in preclinical electrochemotherapy studies, while allowing controlled evaluation of electric field distribution within both tumor and adjacent healthy tissue.

**Figure 3:**
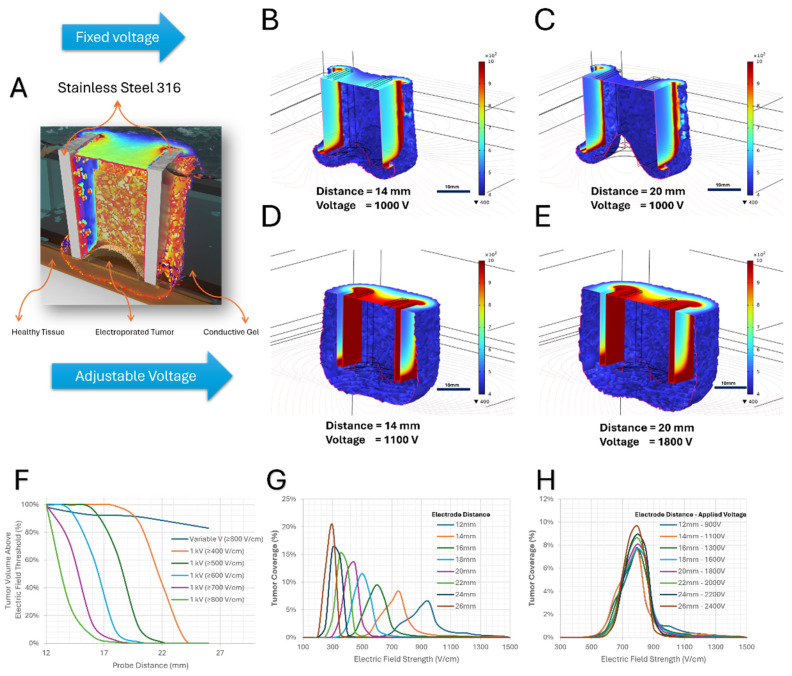
Numerical simulation of electric field distribution generated by variable plate electrodes surrounding a 1 cm tumor embedded in healthy tissue. (**A**) Baseline electric field distribution between two parallel steel plate electrodes. (**B**,**C**) First simulation stage showing electric field distribution for different inter-electrode distances at a fixed applied voltage (1000 V), resulting in non-uniform fields and partial tumor coverage. (**D**,**E**) Second simulation stage employing adjustable voltage in combination with variable electrode spacing, producing a more homogeneous electric field that completely surrounds the tumor volume. Color maps represent electric field magnitude (V/cm), illustrating the improved field uniformity achieved using the variable plate electrode approach. (**F**) Tumor volume fraction exposed to electric fields above the treatment threshold versus probe distance. Under fixed voltage (1 kV), the tumor volume above the threshold decreases rapidly as electrode spacing increases. In contrast, the variable-voltage configuration maintains a more stable tumor coverage by adjusting the applied voltage. (**G**) Distribution of tumor coverage as a function of electric field strength for different electrode distances under a fixed applied voltage of 1 kV. Increasing electrode separation shifts the peak electric field toward lower values, moving a larger portion of the tumor below the electroporation threshold. Additionally, the distribution becomes narrower with increasing distance, indicating a reduction in the spread (standard deviation) of the electric field within the tumor. (**H**) Distribution of tumor coverage as a function of electric field strength for different electrode distances with adjusted applied voltage. By increasing the applied voltage as electrode spacing increases, the mean electric field within the tumor remains approximately constant. Additionally, the distributions become narrower at larger distances, indicating reduced standard deviation and a more uniform electric field within the treated region. An animated version of the electric field distribution corresponding to this figure is available as Supplementary Videos S1 and S2.

Two parallel stainless-steel plate electrodes were positioned on opposite sides of the tumor along a single axis, forming a plate-to-plate electroporation configuration. To investigate the influence of electrode geometry and spacing on electric field uniformity, the lateral dimensions of the electrodes were varied between 1.0 cm and 2.5 cm. In the first stage, the distance between the two plate electrodes was systematically varied while the applied voltage was kept constant, allowing assessment of how electrode spacing alone influences electric field magnitude and spatial distribution within the tumor and surrounding tissue. In the second stage, the electrode distance was adjusted in combination with a variable applied voltage in order to maintain a target electric field strength across the treatment region. Comparison of these two stages demonstrated that, while fixed-voltage configurations led to non-uniform field distributions and incomplete tumor coverage at certain electrode spacings, the variable-voltage approach enabled generation of a more homogeneous electric field that fully surrounded the tumor volume. As illustrated in Figure 3B,C, increasing electrode separation under fixed voltage conditions resulted in pronounced field gradients and localized high-intensity regions near the electrode edges, leaving portions of the tumor exposed to sub-therapeutic field strengths. In contrast, when voltage was adjusted according to electrode spacing (Figure 3D,E), more uniform electric field distribution was achieved throughout the tumor, providing complete and symmetric coverage. These results highlight the critical role of the variable plate electrode design in achieving controlled and uniform electroporation conditions.

The space between the electrodes and the tissue surface was filled with a conductive gel layer to ensure optimal electrical contact and minimize interfacial impedance. As shown in Figure 3A, the presence of the conductive gel facilitated smooth current transfer from the steel plates into the tissue, reduced edge effects at the electrode–tissue interface, and suppressed electric field distortion near the boundaries. This conductive medium promoted uniform current injection and significantly improved electric field homogeneity across the tumor volume. The inclusion of the conductive gel in the simulation reflects realistic experimental conditions and plays a critical role in achieving stable and reproducible electroporation fields. The effect of electrode spacing on tumor coverage was evaluated by analyzing the fraction of tumor volume exposed to electric fields above the treatment threshold. Under a fixed applied voltage of 1 kV, increasing the electrode distance led to a rapid reduction in the tumor volume exceeding the threshold, as the electric field intensity decreased within the tumor. In contrast, adjusting the applied voltage with electrode spacing maintained more consistent tumor coverage (Figure 3F).

Analysis of the electric field distribution showed that increasing electrode separation under fixed voltage shifted the peak field toward lower values and narrowed the distribution, indicating reduced spread of the electric field within the tumor (Figure 3G). When the applied voltage was increased with electrode spacing, the mean electric field remained approximately constant and the distributions became narrower, reflecting a more uniform electric field in the treated region (Figure 3H). Animated visualizations of the electric field distributions are provided in Supplementary Videos S1 and S2.

### 3.4. Vitamin C–Electroporation Interaction in Cell Line Treatment

Numerous investigations have examined the biological activity of vitamin C in cancer models, highlighting its potential as an oxidative stress–modulating and selectively cytotoxic agent at elevated concentrations. In this study, the impact of vitamin C on the viability of MDA-MB-231 breast cancer cells was assessed using the MTT assay. The results demonstrated a clear dose-dependent decline in cell survival, where increasing vitamin C concentrations progressively reduced metabolic activity relative to the untreated controls. Based on the dose–response curve, the IC_50_ of vitamin C was determined to be 15 mM, which is substantially higher than the 1 mM concentration used in the cell line study.

**Figure 4:**
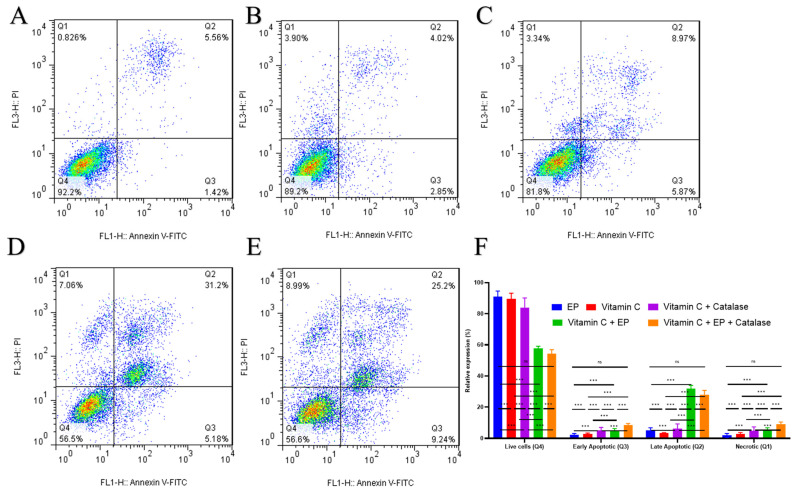
Flow cytometric analysis of apoptosis following treatment. Apoptosis was evaluated using Annexin V–FITC/propidium iodide (PI) staining and analyzed by flow cytometry in five experimental groups: (**A**) Electroporation (EP), (**B**) Vitamin C (1 mM), (**C**) Vitamin C (1 mM) combined with catalase (20 µg/L), (**D**) vitamin C (1 mM) combined with electroporation (VitC + EP) and (**E**) Vitamin C (1 mM) combined with electroporation and catalase (20 µg/L) (VitC + EP + catalase). Representative dot plots of Annexin V–FITC (FL1) versus PI (FL3) staining are shown. In the dot plots, Q4 (Annexin V^−^/PI^−^) represents viable cells, Q3 (Annexin V^+^/PI^−^) represents early apoptotic cells, Q2 (Annexin V^+^/PI^+^) represents late apoptotic/secondary necrotic cells, and Q1 (Annexin V^−^/PI^+^) represents necrotic cells. The percentage of cells in each quadrant is indicated in the respective panels. (**F**) Bar graphs summarize the quantitative analysis of apoptotic cell populations across the five treatment groups. Data are presented as mean ± SD (*n* = 5 per group). Statistical analysis was performed using one-way ANOVA followed by Tukey’s post hoc test, with significance indicated as ns (not significant), *** *p* < 0.001.

To further investigate whether electroporation could potentiate the intracellular action of vitamin C, a single concentration of 1 mM was selected for combined treatment. This concentration was chosen to evaluate whether membrane permeabilization could enhance vitamin C uptake and increase cytotoxicity. When vitamin C was paired with reversible electroporation, a substantial additional decrease in cell viability was detected compared with vitamin C alone. At 1 mM, vitamin C reduced viability to about 90% of the control, whereas the addition of electroporation decreased survival to below 60%. Vitamin C alone produced only a slight decline in viability (Figure 4), and its combination with electroporation resulted in a significant reduction in cell viability (Figure 4D). The catalase (20 µg/L) groups were included to demonstrate that extracellular ROS do not influence the observed effects. As shown in Figure 4C,E, only slight differences were detected between the original groups and the catalase-treated groups.

This result was achieved using a substantially lower dose than Dose 1, with cells exposed to vitamin C for only 15 min. Electroporation significantly enhanced the efficacy of this low dose within this short exposure period. A summary of the flow cytometry data is presented in Figure 4F.

**Figure 5:**
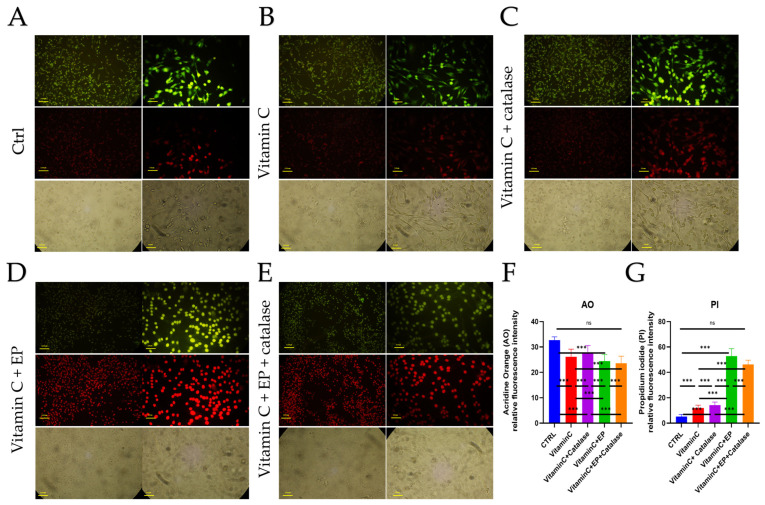
Representative fluorescence microscopy images of acridine orange/propidium iodide (AO/PI) staining in five experimental groups: (**A**) Control (CTRL), (**B**) Vitamin C (1 mM), (**C**) Vitamin C (1 mM) combined with catalase (20 µg/L), (**D**) vitamin C (1 mM) combined with electroporation (VitC + EP) and (**E**) Vitamin C (1 mM) combined with electroporation and catalase (20 µg/L) (VitC + EP + catalase). AO stains viable cells green, while PI stains membrane-compromised (non-viable) cells red. Corresponding bright-field images are shown for morphological reference. (**F**) Quantitative analysis of AO-positive and (**G**) PI-positive cells across groups. Data are presented as mean ± SD (*n* = 5). Statistical analysis was performed using one-way ANOVA followed by Tukey’s post hoc test. Significance is indicated as ns (not significant), *** *p* < 0.001.

As shown in Figure 5, acridine orange/propidium iodide (AO/PI) staining was used to qualitatively assess cell viability and membrane integrity following treatment with vitamin C and electroporation. Representative brightfield images demonstrate preserved cellular morphology in the control (CTRL) group, with cells maintaining their typical adherent shape and intact structure (Figure 5A). Consistent with these observations, AO fluorescence images show predominantly green-stained nuclei, indicating a high proportion of viable or early apoptotic cells, while minimal PI uptake is detected.

In cells treated with vitamin C (1 mM) alone, a modest alteration in cell morphology is observed (Figure 5B), accompanied by a slight increase in PI-positive cells. This finding suggests limited induction of late apoptosis or membrane compromise at this concentration when vitamin C is applied without electroporation. Nevertheless, the majority of cells in this group retain green AO fluorescence, indicating that low-dose vitamin C alone exerts only a mild cytotoxic effect under these conditions.

In contrast, the combined vitamin C and electroporation (VitC + EP) group exhibits a marked increase in red PI fluorescence, as clearly illustrated in Figure 5D. This effect is accompanied by visible morphological disruption in brightfield images, including cell rounding and loss of membrane integrity. The substantial rise in PI-positive cells indicates enhanced late apoptosis or necrotic cell death, highlighting the critical role of electroporation in facilitating intracellular delivery of vitamin C.

Catalase was added to the AO/PI staining groups to verify that extracellular ROS did not influence cell-viability measurements, as demonstrated separately in Figure 5C,E, where catalase showed no significant effect on the AO/PI staining results.

Collectively, the qualitative AO/PI staining results presented in Figure 5F demonstrate that electroporation significantly amplifies the cytotoxic effect of low-dose vitamin C. These observations support the hypothesis that membrane permeabilization enhances transmembrane transport and intracellular accumulation of vitamin C, thereby increasing its anticancer efficacy while maintaining a low extracellular drug concentration.

### 3.5. Intracellular ROS Generation in MDA-MB-231 Cells Following Vitamin C and Electroporation Treatment

**Figure 6:**
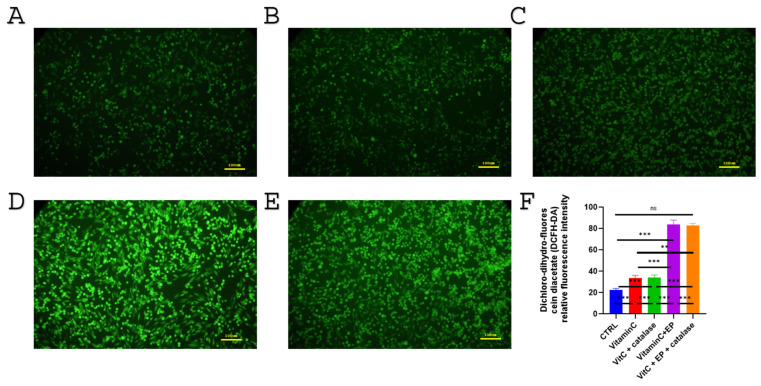
Representative fluorescence microscopy images of intracellular ROS generation in MDA-MB-231 cells following treatment under different conditions. (**A**) Electroporation; (**B**) Vitamin C; (**C**) Vitamin C + catalase; (**D**) Vitamin C + electroporation; (**E**) Vitamin C + catalase + electroporation. Green fluorescence reflects DCFH-DA oxidation and indicates ROS production. (**F**) Quantitative analysis of mean fluorescence intensity demonstrates that vitamin C increases ROS levels and that this effect is further enhanced by electroporation. Data are presented as mean ± SD (*n* = 5). Statistical analysis was performed using one-way ANOVA followed by Tukey’s post hoc test. Significance is indicated as ns (not significant), ** *p* < 0.01 and *** *p* < 0.001.

Intracellular ROS generation in MDA-MB-231 cells was assessed using DCFH-DA fluorescence microscopy. As shown in Figure 6A, the control group exhibited low basal fluorescence, indicating minimal ROS production. Treatment with vitamin C (Figure 6B) and vitamin C combined with catalase (Figure 6C) increased fluorescence intensity compared with the control group, suggesting elevated intracellular ROS levels. Notably, the combination of vitamin C with electroporation (Figure 6D) and vitamin C with catalase plus electroporation (Figure 6E) resulted in a further increase in green fluorescence, indicating enhanced ROS generation following electroporation. Quantitative analysis of fluorescence intensity confirmed that vitamin C induced ROS production and that this effect was significantly amplified when combined with electroporation (Figure 6F).

To evaluate the possible contribution of extracellular ROS during the electroporation process, catalase was added to the PBS treatment solution to enzymatically decompose extracellular H_2_O_2_. Cells were maintained in the PBS-based treatment solution for 15 min, corresponding to the time window during which electroporation-induced membrane pores remain transiently open. This approach allowed comparison of ROS generation in the presence and absence of extracellular H_2_O_2_ removal.

### 3.6. Application of Vitamin C-Assisted ECT in Mouse Model

**Figure 7:**
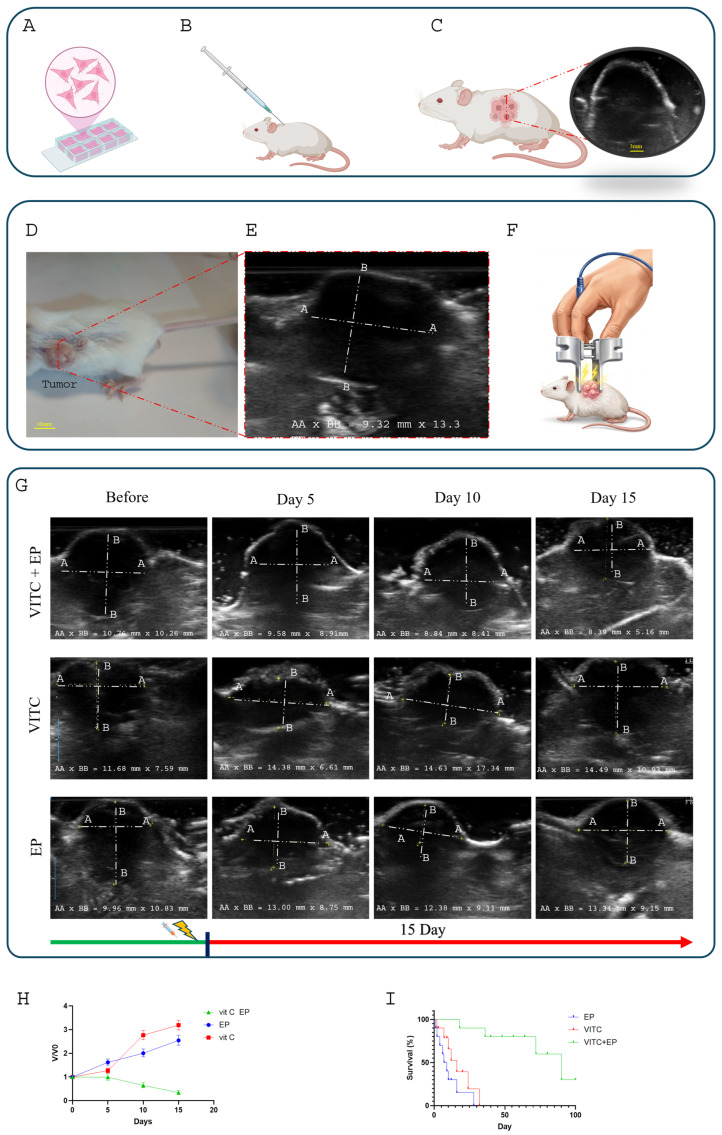
In vivo evaluation of tumor treatment and survival analysis following electroporation therapy (**A**) 4T1 breast cancer cells were cultured in RPMI-1640 medium. (**B**) A suspension of 1 × 10^6^ 4T1 cells in 0.1 mL phosphate-buffered saline (PBS) was subcutaneously injected into the flank region of each mouse. (**C**) Tumors were assessed for suitability for treatment, after which mice were randomly allocated into three experimental groups. (**D**,**E**) Tumor size was assessed using ultrasound imaging, and tumor volume was calculated at the beginning of the treatment. (**F**) The designated treatment protocol was applied to the tumor-bearing mice according to the assigned experimental group. (**G**) Tumor size was monitored every 5 days using ultrasound imaging to evaluate treatment response and tumor growth dynamics. (**H**) Relative tumor volume (V/V_0_) was evaluated over time and normalized to the tumor volume at day 0. Tumor growth was monitored in the treatment groups receiving vitamin C (Vit C), electroporation (EP), and the combined Vit C + EP therapy. (**I**) Kaplan–Meier survival analysis of tumor-bearing mice. Each group consisted of *n* = 10 mice. Survival differences between groups were analyzed using the log-rank test (*p* < 0.0001).

The in vivo evaluation of vitamin C–enhanced electrochemotherapy (ECT) was conducted using BALB/c mice bearing 4T1 tumors located in the flank region (Figure 7A). The 4T1 tumor model was selected because it closely mimics several pathological characteristics of stage IV human breast cancer (Figure 7B). Prior to treatment, tumor size was assessed using ultrasound imaging, and the tumor volume was estimated using an ellipsoid-based formula (Figure 7C). Before initiating the therapeutic procedure, the mice were anesthetized, after which electrochemotherapy treatment was performed (Figure 7E). Figure 7F demonstrates the application of the electrode in the electrochemotherapy treatment of a tumor that has been induced subcutaneously in a BALB/C mouse model. In this study, mice were assigned to three experimental groups to investigate the effects of vitamin C and electroporation—both individually and in combination. The control group (CTRL) received only reversible electroporation stimulation; this group is referred to as the EP group. Finally, the combination therapy groups, vitamin C followed by electroporation to determine whether electroporation enhances vitamin C uptake and improves therapeutic efficacy. After treatment, the animals were carefully monitored and tumor progression was evaluated using ultrasound imaging for a period of 15 days. The sonographic measurements of tumor volume are presented in Figure 7G. In the control group, a continuous increase in tumor size was observed. By day 15, ultrasound images revealed that the tumors in the control mice had a larger cross-sectional area compared with their size before treatment.

In contrast, mice treated with the vitamin C–assisted ECT showed a reduction in tumor volume following therapy. These findings indicate that the combination of electroporation with vitamin C led to tumor regression when compared with both the untreated control group and the group receiving vitamin C alone.

To further assess these effects, a quantitative analysis was conducted. Figure 7H illustrates the temporal changes in the relative tumor volume over the 15-day observation period, normalized to the baseline measurements obtained on day 0. Analysis of tumor progression over the 15-day period revealed clear differences among the three experimental groups. In the vitamin C cohort, the tumor volume increased steadily, reaching approximately 3.17-fold its initial size, while the EP group showed a similar upward trend, expanding to nearly 2.54-fold of its baseline value. In contrast, the vitamin C-EP (VitC + EP) group showed a significant therapeutic effect, with tumor volume reducing to approximately 0.34-fold, indicating robust tumor growth suppression as anticipated.

These findings strongly support electroporation as an effective strategy for improving vitamin C-mediated antitumor activity.

**Figure 8:**
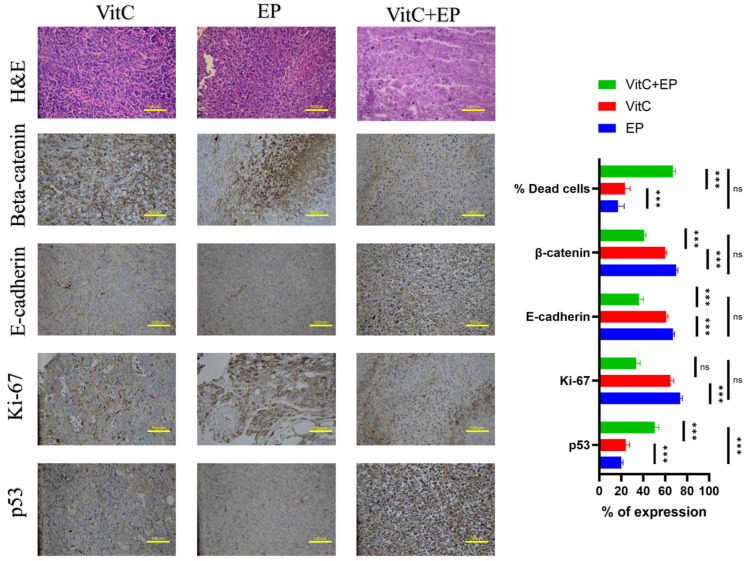
Histological and immunohistochemical evaluation of tumor tissues following treatment. Representative hematoxylin and eosin (H&E) staining and immunohistochemical (IHC) analysis of β-catenin, E-cadherin, Ki-67, and p53 in tumor sections obtained from three experimental groups: electroporation (EP, control), vitamin C (VitC), and combined vitamin C with electroporation (VitC + EP). Quantification of IHC staining was performed by calculating the percentage of positively stained cells from multiple randomly selected microscopic fields per section. Data are presented as mean ± SD (*n* = 5 per group). Ten to fifteen 200× fields-of-view (FOV) were randomly recorded and analyzed. Scale bar = 100 µm. Statistical analysis was performed using one-way ANOVA followed by Tukey’s post hoc test, with significance indicated as ns (not significant), *** *p* < 0.001.

The H&E-stained tissue sections from specimens treated with ascorbic-acid–assisted electrochemotherapy show evidence of necrosis, apoptosis, and reduced cellularity (Figure 8). Histological and immunohistochemical evaluations showed that both the EP (control) group and the vitamin C group exhibited tissue architecture and marker expression patterns consistent with normal tissue, with no notable pathological alterations. H&E staining, as well as β-catenin, E-cadherin, Ki-67, and p53 immunostaining, remained largely comparable between these two groups.

In contrast, the VitC + EP group demonstrated marked deviations from normal morphology. H&E sections showed clear structural disruption and areas of cell loss, while immunostaining revealed reduced β-catenin and E-cadherin expression, along with notable changes in Ki-67 and p53 patterns. These findings indicate that the combined treatment induced significant tissue alterations not observed in the EP or vitamin C groups. Overall, the combination treatment produced the greatest cytotoxic impact among the three groups.

To further evaluate the safety profile of the treatment, histopathological analysis of major organs was performed. Lung and spleen tissues collected from treated mice were examined using hematoxylin and eosin (H&E) staining to assess potential systemic toxicity. As shown in Supplementary Figure S1, no evident structural abnormalities, inflammatory infiltration, or tissue damage were observed in these organs compared with the control group. These findings suggest that the treatment did not induce detectable pathological alterations in the examined tissues under the experimental conditions. The corresponding histological images are provided in the Supplementary Materials as Figure S1.

Although the combined treatment produced greater antitumor effects than either intervention alone, a formal quantitative assessment of drug interaction using the Chou–Talalay Combination Index was not performed. Therefore, the results should be interpreted as an enhanced combined effect rather than definitive pharmacological synergy.

## 4. Conclusions

This study demonstrates that electroporation markedly enhances the therapeutic efficacy of vitamin C in both cellular and tissue models. Although reversible electroporation alone (EP group) and vitamin C treatment alone produced minimal structural or molecular alterations—showing profiles similar to normal tissue—the combination of vitamin C with electroporation resulted in significant cytotoxicity and tissue disruption. Flow cytometry confirmed a substantial increase in late apoptotic and necrotic populations only in the combined treatment group, while histological and immunohistochemical analyses (H&E, β-catenin, E-cadherin, Ki-67, and p53) revealed pronounced morphological damage, reduced proliferation, and elevated stress responses. These effects occurred despite the use of a relatively low dose of vitamin C and a short exposure time, indicating that electroporation effectively potentiates intracellular delivery and amplifies its biological activity. Although vitamin C typically acts as an antioxidant at physiological concentrations, it can shift to a pro-oxidant role under pharmacologic conditions or in the presence of catalytic transition metals. In such environments—particularly within cancer cells, which often contain elevated levels of labile iron—ascorbate can generate hydrogen peroxide and other reactive oxygen species, thereby intensifying oxidative stress. The enhanced intracellular uptake mediated by electroporation likely magnifies this pro-oxidant effect, contributing to the pronounced cytotoxicity observed in the combined treatment group.

## Figures and Tables

**Table 1:** Electrical conductivity and relative permittivity values assigned to biological tissues and materials used in the computational model, including 4T1 tumor, healthy tissue, air, PLA, conductive hydrogels, and stainless steel (316), based on literature-reported parameters.

Medium	Electrical Conductivity (S/m)	Relative Permittivity
4T1 tumor	0.4	4 × 10^7^
Healthy tissue	0.3	4 × 10^8^
Air	1 × 10^−12^	1
PLA	4.2 × 10^−12^	2.724
Conductive Hydrogels	1	70
Steel 316	1.35 × 10^6^	1

## Data Availability

The data supporting the findings of this study are available from the corresponding author upon request. Numerical simulation data, in vitro experimental results, and in vivo tumor measurement data were generated specifically for this study. Due to ethical restrictions related to animal experiments and institutional policies, the complete datasets are not publicly available.

## Supplementary Materials

The following supporting information can be downloaded at: https://www.mdpi.com/article/10.3390/biomedicines14040936/s1, Figure S1: Histopathological evaluation of lung and spleen tissues from treated mice using H&E staining. No structural abnormalities, inflammatory infiltration, or tissue damage were observed compared with the control group, indicating the absence of detectable systemic toxicity under the experimental conditions.; Video S1: Animation illustrating the distribution of tumor coverage as a function of electric field strength at different electrode distances under a constant 1 kV applied voltage.; Video S2: Animation illustrating the distribution of tumor coverage as a function of electric field strength when the applied voltage is adjusted to compensate for increasing electrode distance, maintaining a comparable mean electric field.

## Abbreviations

The following abbreviations are used in this manuscript:

AO/PI	Acridine Orange/Propidium Iodide
Asc	Vitamin C
BALB/c	Bagg Albino Laboratory-Bred mouse strain
COMSOL	COMSOL Multiphysics^®^
DHA	Dehydrovitamin C
ECT	Electrochemotherapy
EP	Electroporation
FBS	Fetal Bovine Serum
FDM	Fused Deposition Modeling
GAPDH	Glyceraldehyde-3-Phosphate Dehydrogenase
GenAI	Generative Artificial Intelligence
GLUT	Glucose Transporter
GSH	Glutathione
H&E	Hematoxylin and Eosin
H_2_O_2_	Hydrogen Peroxide
IVC	Intravenous Vitamin C
MTT	3-(4,5-Dimethylthiazol-2-yl)-2,5-Diphenyltetrazolium Bromide
OC	Oral vitamin C
PBS	Phosphate-Buffered Saline
PLA	Polylactic Acid
ROS	Reactive Oxygen Species
RPM	Revolutions Per Minute
SD	Standard Deviation
SVCT	Sodium-Dependent Vitamin C Transporter
SVCT1	Sodium-Dependent Vitamin C Transporter 1
SVCT2	Sodium-Dependent Vitamin C Transporter 2
TNBC	Triple-Negative Breast Cancer
VitC	Vitamin C
