# Supplementary figures and images for: Low-Dose Vitamin C-Based Electroporation of Solid Tumors: A New Area in Non-Cytotoxic Electrochemotherapy
Source: Biomedicines. 2026 Apr 20;14(4):936. doi: 10.3390/biomedicines14040936 (PMC13113664; doi:10.3390/biomedicines14040936)

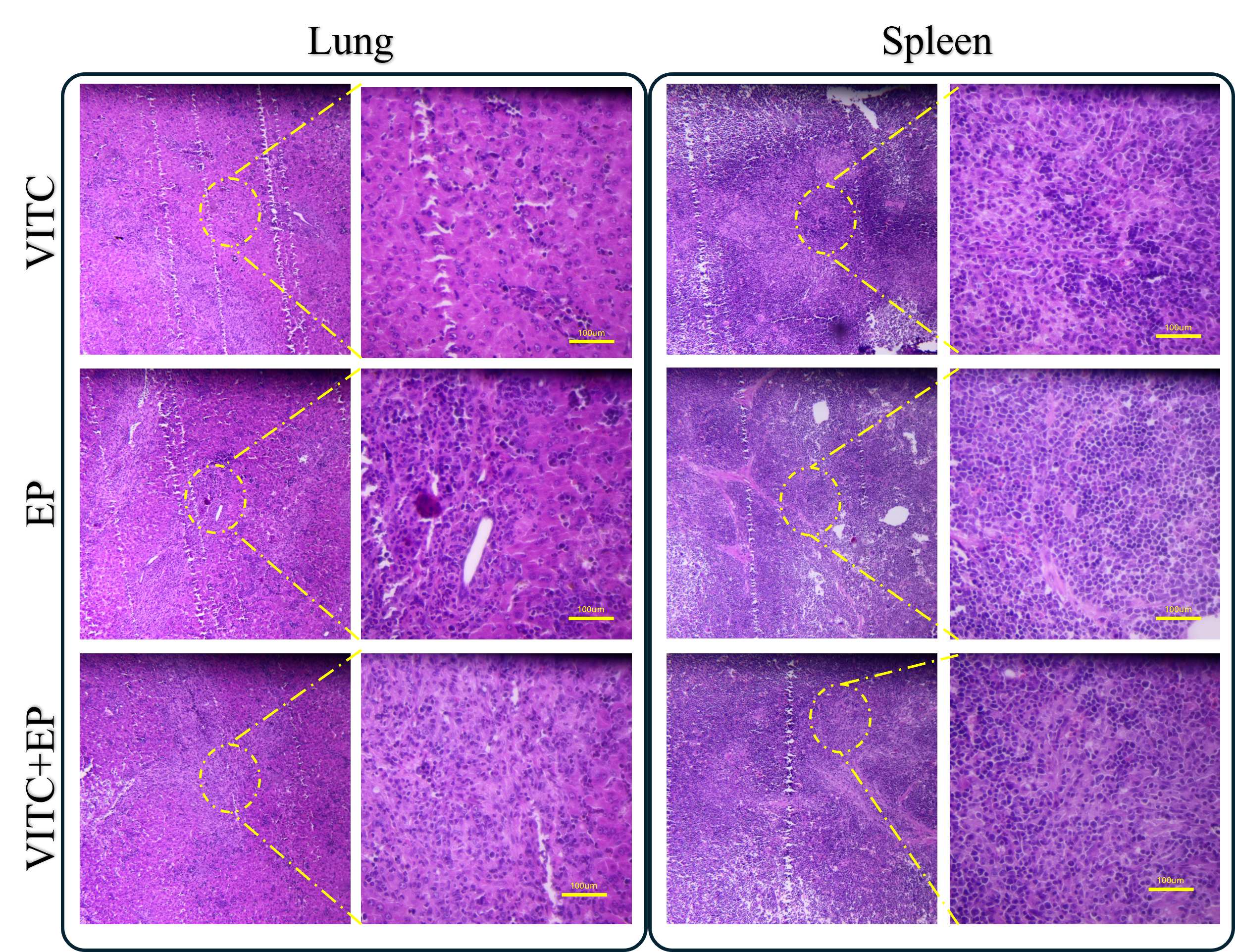

Supplement: Supplementary file 1 [file biomedicines-14-00936-s001.zip › fig S1.tif]

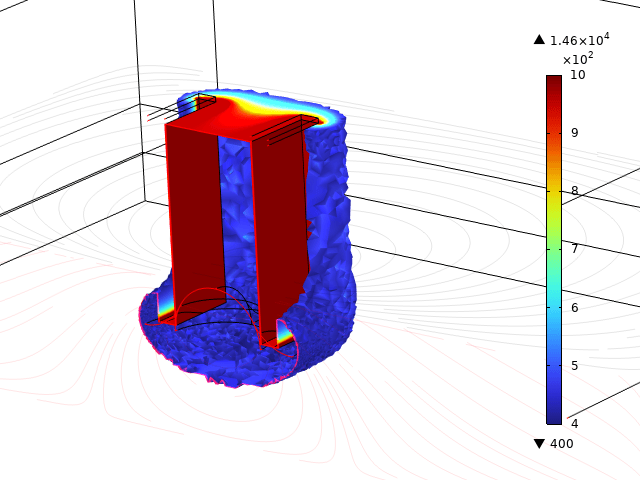

Supplement: Supplementary file 1 [file biomedicines-14-00936-s001.zip › Video S1.gif]

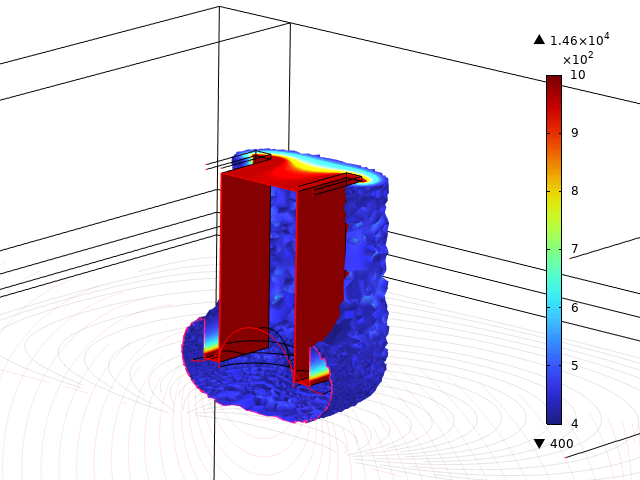

Supplement: Supplementary file 1 [file biomedicines-14-00936-s001.zip › Video S2.gif]
